# The Diversity of the *Limnohabitans* Genus, an Important Group of Freshwater Bacterioplankton, by Characterization of 35 Isolated Strains

**DOI:** 10.1371/journal.pone.0058209

**Published:** 2013-03-07

**Authors:** Vojtěch Kasalický, Jan Jezbera, Martin W. Hahn, Karel Šimek

**Affiliations:** 1 Faculty of Sciences, University of South Bohemia, České Budějovice, Czech Republic; 2 Biology Centre of the ASCR v.v.i., Institute of Hydrobiology, České Budějovice, Czech Republic; 3 Research Institute for Limnology, University of Innsbruck, Innsbruck, Austria; J. Craig Venter Institute, United States of America

## Abstract

Bacteria of the genus *Limnohabitans*, more precisely the R-BT lineage, have a prominent role in freshwater bacterioplankton communities due to their high rates of substrate uptake and growth, growth on algal-derived substrates and high mortality rates from bacterivory. Moreover, due to their generally larger mean cell volume, compared to typical bacterioplankton cells, they contribute over-proportionally to total bacterioplankton biomass. Here we present genetic, morphological and ecophysiological properties of 35 bacterial strains affiliated with the *Limnohabitans* genus newly isolated from 11 non-acidic European freshwater habitats. The low genetic diversity indicated by the previous studies using the ribosomal SSU gene highly contrasted with the surprisingly rich morphologies and different patterns in substrate utilization of isolated strains. Therefore, the intergenic spacer between 16S and 23S rRNA genes was successfully tested as a fine-scale marker to delineate individual lineages and even genotypes. For further studies, we propose the division of the *Limnohabitans* genus into five lineages (provisionally named as LimA, LimB, LimC, LimD and LimE) and also additional sublineages within the most diversified lineage LimC. Such a delineation is supported by the morphology of isolated strains which predetermine large differences in their ecology.

## Introduction

*Betaproteobacteria* frequently belong to the most abundant members of freshwater bacterioplankton [1], [2]. It is assumed that only seven [3] main lineages are present in freshwater habitats worldwide. The genus *Limnohabitans* (*Betaproteobacteria, Comamonadaceae*) has been recently established [4] as a group of environmentally important “not-easily cultivable” freshwater bacteria from the BetI lineage [5]. The genus is currently composed of four described *Limnohabitans* species [4], [6], [7] and four lineages (Lhab-A1 to A4) that have been proposed within the genus [3]. Two species *L. planktonicus* and *L. parvus* [7], belong to the R-BT lineage, targeted by the R-BT065 FISH (fluorescence *in situ* hybridization) probe [8]. Just recently, a large database containing environmental sequences from R-BT group has been established [3].

The bacteria from the R-BT lineage are known to inhabit a broad range of freshwater habitats within at least three continents and can constitute up to 30% of free-living bacteria in freshwater systems [5], [9], [10], [11]. It has been shown that they strongly prefer non-acidic habitats and their abundance in low pH habitats is usually negligible [11]. In lakes, they inhabit the neuston [12], the epilimnion [8], and the hypolimnion [13], [14], indicating their capabilities to live in both oxic and anoxic environments [13].

The R-BT lineage is known to be represented by phylotypes with opportunistic strategies [15], [16]. The R-BTs are characterized by a high percentage of cells incorporating leucine [13], [17], [18], [19] and glucose [13], whereas low uptake rates were measured for thymidine [17], [19] and acetate [13] and no uptake for the incorporation of 4-hydroxybenzoic acid [13]. Notably, the R-BT bacteria displayed the highest growth rate among major bacterioplankton lineages, comparable to growth rates of small heterotrophic nanoflagellates under *in situ* conditions [20]. Interestingly, experimentally manipulated grazing pressure markedly accelerated growth of R-BT065 targeted bacteria [21], which were moreover preferentially ingested by these flagellates [22]. Further, these results were complemented with a specific study examining niche separation in two closely related species of *L. parvus* and *L. planktonicus* [23], based on their size, growth capabilities, vulnerability to protozoan grazing, and virus infection.

The predominant natural source of substrates for the R-BTs seems to be autochthonous algal-derived organic material [23], [24], [25]. Notably, growth of *L. parvus* and *L. planktonicus* on algal exudates as a sole dissolved organic carbon (DOC) source has just been confirmed [26]. Products of the photolysis of dissolved organic matter have also been suggested as an important additional source of substrates for these bacteria [12], [27]. Just recently, a complete photosynthesis gene cluster, RuBisCO and CO dehydrogenase genes have been found in genomes of two *Limnohabitans* strains Rim28 and Rim47 [28]. However, as yet any experimental confirmations of the genes expression into corresponding and detectable metabolic traits are missing.

In contrast to the considerable information on the ecophysiology of the R-BT group available, we have almost no knowledge on the ecology of the other two described *Limnohabitans* species *L*. *curvus* and *L. australis* [4], [6], since no specific FISH probes are currently available to follow their *in situ* population dynamics. Perhaps, a more specific immuno-staining essays could be a way to overcome the problem with the limited resolution of currently available FISH probes.

The wide range of pH occupied (4.9–9.1) [11] in combination with the marked ecophysiological capabilities of R-BT bacteria (see above) suggests a large microdiversity within the cluster. However, existing 16S rRNA gene sequences show more than 96% identity, suggesting either that genetic diversity is low or that 16S rRNA is an inappropriate target for diversity assessment. To distinguish between these two possibilities, we established comprehensive sets of molecular and ecological data in a polyphasic approach building on additional representative strains isolated from the *Limnohabitans* genus and the R-BT lineage.

In this paper, we characterize ecophysiological patterns and analyze the phylogeny and morphology of 35 newly isolated strains affiliated within the *Limnohabitans* genus. The aims of the presented study were: (i) to examine the diversity within the *Limnohabitans* genus by sequencing of the 16S rRNA gene and the IGS1 loci (the intergenic spacer between 16S and 23S rRNA genes) of the newly isolated *Limnohabitans* strains and characterization of phylogenetically distinct lineages within the genus, (ii) to investigate metabolic capabilities and morphological and size-related characteristics of the isolated strains and to interpret these phenotypic traits regarding potential differences in ecological adaptations, and (iii) to estimate the contribution of R-BT bacteria to total abundance and biomass of bacterioplankton in seven ecologically contrasting habitats.

## Results

### Growth Abilities and Morphological Traits of Isolated Strains

Thirty-five bacterial strains affiliated within the *Limnohabitans* genus were isolated from 12 ecologically diverse freshwater habitats (Table 1). Seven habitats can be assigned to the category “Fishponds and reservoirs”, four to “Alkaline lakes”, one to “Small shallow ponds” as predefined by Šimek et al. [23]. However, we failed to isolate *Limnohabitans* strains from low pH habitats such as “Humic lakes and ponds” or “Acidified lakes”. Usually, one or two R-BT-positive wells were present among 100 to 150 wells displaying turbidity, however the proportion of *Betaproteobacteria*-positive wells was always much higher and varied broadly (data not shown).

**Table 1:** Characteristics of freshwater habitats from which *Limnohabitans* spp. strains were isolated.

Habitat	No. of isolates	Habitat characteristics	Surface area (ha)	Geographic coordinates	Country	Trophic status	pH	Conductivity (µS cm^−1^)
Klíčava reservoir	12	reservoir for drinking water supply	74.1	50°3′58′′N, 13°55′55′′E	Czech Republic	mesoeutrophic	8.9	452
Římov reservoir	8	reservoir for drinking water supply	206	48°50′N, 14°29′E	Czech Republic	mesoeutrophic	7–8	100–120
Bagr pond	3	shallow urban pond with a concretebottom	1	48°58′17′′N, 14°27′23′′E	Czech Republic	eutrophic	7–8	300–400
Gosau 3	2	small shallow montane lake	0.04	47°35′23′′N, 13°34′13′′E	Austria	oligomesotrophic	7.5	493
Lake Loosdrecht	2	large shallow peat lake	980	52°12′15.91′′N, 5°4′52.58′′E	Netherlands	eutrophic	8.0	420
Lužnice pond T6	2	small deep pond in Lužnice riverbed	0.01	48°50′0.453′′N, 14°55′40.324′′E	Czech Republic	eutrophic	6.8	200
Seepromenade	2	small shallow urban pond in Mondsee	0.02	47°51′06.36′′N, 13°21′04.34′′E	Austria	eutrophic	7.4	341
Hintersee	1	deep submontane lake in prealpine region	82	47°44′49′′N, 13°14′59′′E	Austria	oligomesotrophic	8.5	249
Mondsee	1	deep submontane lake in prealpineregion	1378	47°50′N, 13°20′E	Austria	oligomesotrophic	8.3	323
Nový u Cepu pond	1	shallow fishpond	10.5	48°55′16.912′′N, 14°49′52.806′′E	Czech Republic	hypertrophic	7	211
Wiestalstausee	1	reservoir in prealpine region	70	47°44′47.46′′N, 13°10′13.96′′E	Austria	oligomesotrophic	8.3	275

**Figure 1:**
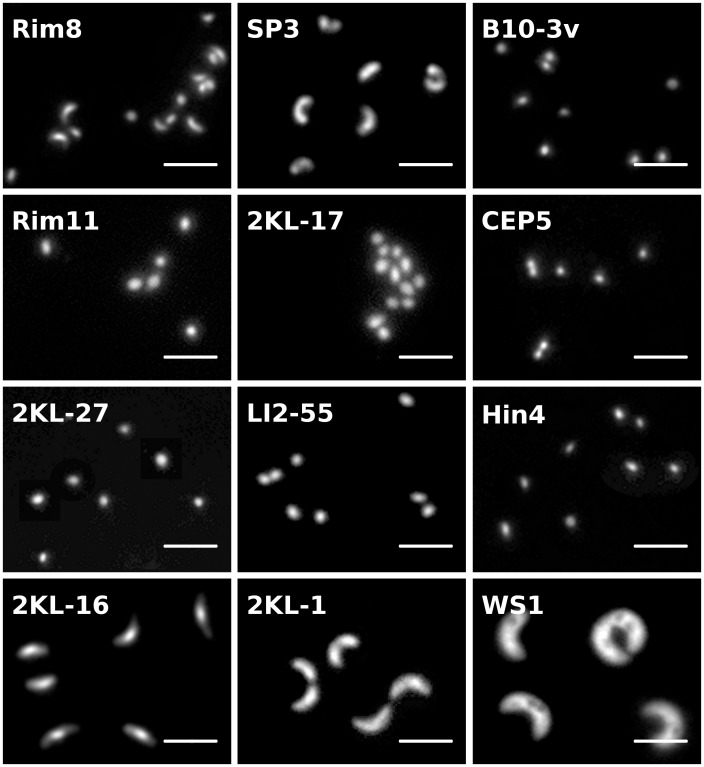
Basic morphotypes of isolated *Limnohabitans* spp. strains. (A, B) Lineage LimA, (C) lineage LimE, (D) lineage LimB, (E–L) lineage LimC – strains 2KL-17 (unaffiliated), CEP5 (LimC3), 2KL-27 (LimC5), LI2-55 (LimC2), Hin4 (LimC4), 2KL-16 (LimC1), 2KL-1 (unaffiliated) and WS1 (LimC6). The strain-specific codes refer to the codes assigned to isolates in the overview Table 2. Microphotographs, 1000×magnification, scale bar represents 2 µm.

All isolated strains were screened microscopically for their shape and size at the end of the acclimation procedure and during the purification, and regularly checked by FISH with the R-BT065 and the Bet42a probes. The isolated strain morphologies were: coccoid, ovoid or short-rod (20 strains), rod (1 strain), curved rod (2 strains), solenoid (8 strains) or large solenoid/C-shaped morphology (5 strains, see Fig. 1). Cell sizes spanned over a wide range of sizes from 0.4 µm-diameter of cocci up to 5 µm in length of curved rods (for details see Table 2).

**Table 2:** The origin and morphological characteristics of isolated strains *Limnohabitans* spp.

			Morphology		
Strain	Habitat	Lineage/sublineage	Cell length (µm)	Cell volume (µm^3^)	Shape
B9-3	Bagr pond	LimE	0.5–1.2	0.04–0.11	ovoid
B10-3v	Bagr pond	LimE	0.5–1.1	0.03–0.06	solenoid
B22-3vk	Bagr pond	LimC4	0.6–1.0	0.05–0.10	short rods
G3-2	Gosau 3	LimC	0.4–0.7	0.04–0.07	coccoid
G3-3	Gosau 3	LimC6	2.1–3.0	0.30–0.52	large solenoid
Hin4	Hintersee	LimC4	0.4–0.6	0.02–0.04	short rods
KL1	Klíčava res.	LimB	0.5–1	nd	short rods
KL5	Klíčava res.	LimC5	0.7–1.0	0.05–0.13	ovoid
KL6	Klíčava res.	LimC	0.5–0.8	0.04–0.07	short rods
KL6S	Klíčava res.	LimA	0.8–1.1	0.06–0.18	solenoid
2KL-1	Klíčava res.	LimC	0.8–1.4	0.08–0.21	solenoid
2KL-3	Klíčava res.	LimC6	2.3–3.4	0.41–0.78	large solenoid
2KL-5	Klíčava res.	LimC	0.5–1.0	0.04–0.13	short rods
2KL-7	Klíčava res.	LimC6	2.7–3.9	0.35–0.83	large solenoid
2KL-15	Klíčava res.	LimB	0.4–0.6	0.02–0.04	coccoid
2KL-16	Klíčava res.	LimC1	1.4–2.2	0.12–0.19	curved rods
2KL-17	Klíčava res.	LimC	0.4–0.7	0.04–0.06	coccoid
2KL-27	Klíčava res.	LimC5	0.5–0.7	0.04–0.07	coccoid
LF5-52	Lake Loosdrecht	LimA	0.6–0.9	0.05–0.09	solenoid
LJ2-35	Lake Loosdrecht	LimC2	0.4–0.6	0.04–0.06	coccoid
Mo2-6	Lake Mondsee	LimC	0.3–0.6	0.02–0.04	coccoid
T6-5	Lužnice pond T6	LimC	1.7–3.1	0.31–0.90	curved rods
T6-20	Lužnice pond T6	LimC3	0.6–0.9	0.04–0.07	coccoid
CEP 5	Nový u Cepu fishpond	LimC3	0.4–0.7	0.03–0.05	coccoid
15K	Římov res.	LimC4	0.4–0.7	0.02–0.04	ovoid
Rim6	Římov res.	LimA	0.9–1.3	0.12–0.25	solenoid
Rim8	Římov res.	LimA	0.5–0.7	0.03–0.05	solenoid
Rim11	Římov res.	LimB	0.5–0.8	0.03–0.05	short rods
Rim28	Římov res.	LimC	0.4–0.6	0.03–0.04	coccoid
Rim42	Římov res.	LimC1	0.6–0.9	0.04–0.08	rods
Rim47	Římov res.	LimC4	0.5–0.7	0.04–0.06	coccoid
VIII-A6	Římov res.	LimC2	0.4–0.6	0.03–0.05	short rods
SP2	Seepromenade	LimC6	2.1–3.0	0.30–0.52	large solenoid
SP3	Seepromenade	LimA	0.7–1.1	0.06–0.10	solenoid
WS1	Wiestalstausee	LimC6	2.1–3.0	0.43–0.68	large solenoid

Gray images of cells were taken with Olympus BX-60 and CCD camera. Note that shape classifications are only subjective.

**Figure 2:**
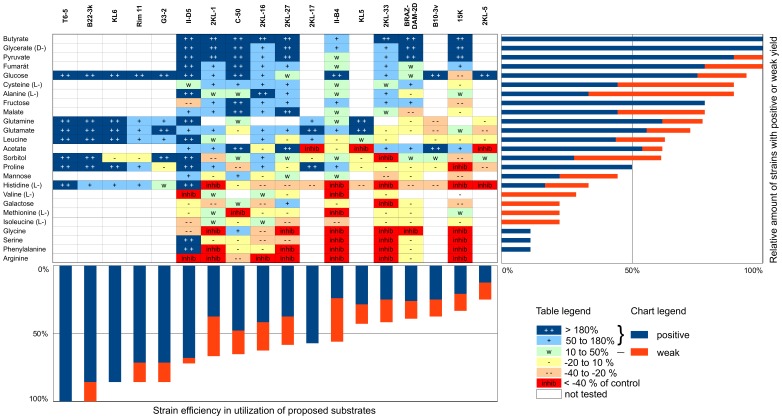
Metabolic characteristics of newly isolated *Limnohabitans* spp. and described species. The characteristics of *L. australis* strain MWH-BRAZ-DAM-2D, *L. curvus* MWH-C5, *L. parvus* II-B4 and *L. planktonicus* II-D5 were taken from [4], [6] and [7], respectively. Biomass increase of strains was scored as++(highly positive, >180% of control treatments),+(positive, 50–180%), w (weak, 10–50%), − (no growth, -20–10%), – (light inhibition, −40–−20%) and inhib (severe inhibition,<-40%). Strains and substrates are sorted according to their relative number of hits when growth growth was observed (<20% biomass increase), i.e. more opportunist on the left and more specialist on the right, and more preferred substrate on the top and less on the bottom.

We investigated abilities of selected strains to utilize different substrates added into the diluted NSY medium (Fig. 2). In total, 18 substrates were utilized by at least one strain (with 4 of them only weakly). The most widely accepted substrates were simple organic acids (butyric, glyceric, pyruvic, fumaric and malic), monosaccharides (glucose and fructose) and some aminoacids (L-alanine, L-cysteine, glutamine and glutamate). Surprisingly, leucine addition induced biomass increases of only half of the tested strains. In contrast, many amino-acid-amended treatments (arginine, phenylalanine, serine, glycine, isoleucine, methionine, valine and histidine) resulted in inhibitory effects on growth of the strains.

Each tested strain showed a biomasss increase for a different spectrum of substrates offered (Fig. 2). For instance, 8 strains yielded significantly higher biomass when grown on more than half of tested substrates whereas only 6 strains could not use more than a half. Eight *Limnohabitans* strains utilized acetate (one weakly) and 7 strains fructose. In contrast, six strains (from 16 tested) were unable to grow on leucine.

### Genetic Diversity and Proposed Division of the Limnohabitans Genus

Almost complete sequences of 16S rRNA genes (1435–1440 bp) and complete sequences of IGS1 regions (648–771 bp, including 2 tRNAs – Ile and Ala) were obtained for all isolated strains. In addition, complete IGS1 sequences were obtained for *L. curvus* MWH-C5^T^, *L. australis* MWH-BRAZ-DAM2D^T^, *L. parvus* II-B4^T^, *L. planktonicus* II-D5^T^, *Rhodoferax fermentans* FR2^T^ and *Curvibacter gracilis* 7-1^T^. The similarity of 16S rRNA gene and IGS1 sequences of isolated *Limnohabitans* strains are >97% and >81% respectively (for more information see Tables S1 and S2 in File S1).

**Figure 3:**
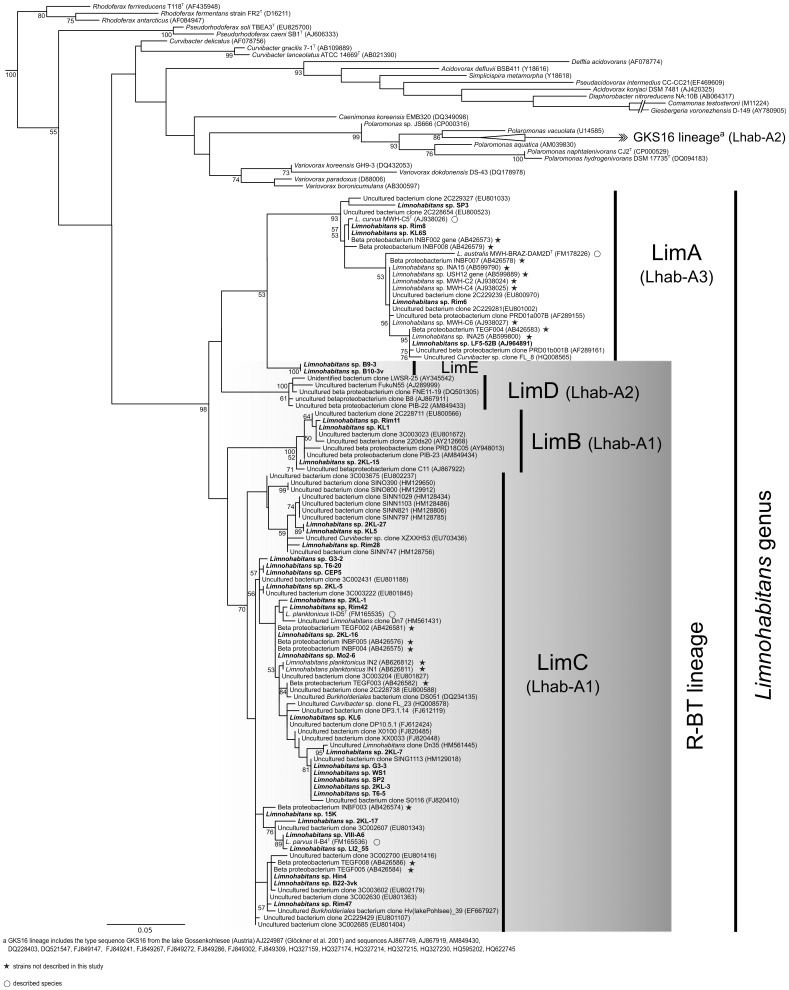
Phylogenetic tree of isolated *Limnohabitans* spp. strains, environmental clones and described species based on 16S rRNA gene. GKS16 cluster is composed of the homonymous clone and other 19 environmental sequences. The consensus tree was constructed by Bayesian algorithm with 8 million generations, when 2000 trees were removed as burnin. The scale bar correspond to 50 base substitutions per 100 nucleotide positions. Bootstrap values for Bayesian probability at the branching points are given. The tree was rooted by *Polynucleobacter necessarius* subsp. *asymbioticus*, *Ralstonia eutropha* and *Herbaspirillum putei*. Detailed description of used dataset is available in Table S3 in File S1.

**Figure 4:**
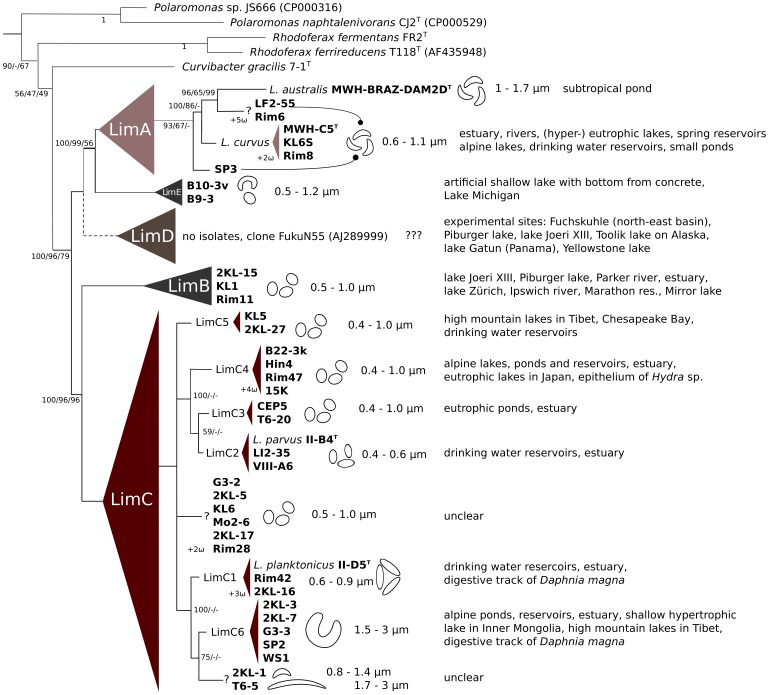
Microdiversity of *Limnohabitans* genus based on 40 isolated strains. The simplified phylogeny schema was build on analyses of 16S rRNA gene and IGS1 sequences. The phylogeny was constructed on the base of Bayesian algorithm with 5 million generations, when 1000 trees were removed as burnin. Bootstrap values for Bayesian probability/Maximum Parsimony/Maximum Likelihood at the branching points are given. The tree was rooted by *Polynucleobacter necessarius* subsp. *asymbioticus*. Symbol “ω” with a number stands as reference for isolated strains obtained by K. Watanabe. Question marks stands for polyphyletic groups of strains with similar morphologies. Listed habitats originate from GenBank/EMBL sequence databases.

Phylogenetic analysis of the 16S rRNA gene sequences, including validly described species and environmental samples, supported the affiliation of the isolated strains within the genus *Limnohabitans* (Fig. 3). Five main lineages (provisionally named LimA, LimB, LimC, LimD and LimE) were consistently observed in phylogenetic trees constructed using different algorithms (NJ, MP, ML, bayesian). The IGS1 phylogeny further confirmed the phylogenetic grouping within the genus *Limnohabitans* presented in this paper (Fig. 4).

In following paragraphs, we would like to describe subgenus-like groups of strains. We avoid of using the term “tribe” as it was introduced by Newton et al. [3] and used by Eiler et al. [29], because of its taxonomical meaning (a group of genera), which is inappropriate for the description of phylogenetic groups within a genus. Since we want to respect the taxonomical code, the proposed hierarchical naming structure (phylum/lineage/clade/tribe) by Newton and coworkers is not sufficiently deep. While another annotation is not available in the Bacteriological Code at the moment, in following lines we will use the term lineage/sublineage for groups of strains or clones within the genus. It is important to note that the terms for subgenus-like groups in the Botanical Taxonomical Code – “section” and “series”, are not recommended by the members of the Judicial Commission of the ICSP (P. Kämpfer and B.J. Tindall, personal communication).

**Lineage LimA** (identical to lineage Lhab-A3 in [3]) is the only group within the genus which does not possess the discriminative sequence 5′- GTT GCC CCC TCT ACC GTT -3′ matching the R-BT065 probe, and consequently their members remain “invisible” by using this probe. Two already described species L *curvus* and *L. australis*, [4], [6] and 5 newly isolated strains are affiliated within this lineage. All 7 strains are morphologically similar, of a solenoid shape (Fig. 1A, 1B and [4], [6]). The 5 new members were isolated from 4 different habitats and they clustered together with other related cultivated strains and environmental sequences available in GenBank a well-separated lineage within the *Limnohabitans* genus. The similarity within the lineage is >98% on 16S rRNA gene and >89% on IGS1 sequence. The new strains KL6S and Rim8 isolated from different habitats (Tables 1 and 2), shared both sequences identical with strain *L. curvus* MWH-C5^T^, thus they most probably represent the same species. All the phylogenetic algorithms used suggested a separation of the strain *L. australis* MWH-BRAZ-DAM2D^T^ vis-a-vis other isolated strains and environmental sequences.

Phylogenetic analyses of both 16S rRNA and IGS1 genes of isolated strains indicate that the large Lhab-A1 lineage [3] is consistently separated into two closely related lineages. We propose to call the lineages LimB and LimC. **Lineage LimB** is represented by three newly introduced strains (Fig. 1B, C, Table 2) and also contains environmental sequences originating from lakes, rivers and estuaries in Switzerland, Austria, Germany, China and 7 states in the USA (see Table S3 in File S1). The strains within the lineage share similarities of their 16S rRNA gene >99.5% and of their IGS1 sequence >89.9%. The new strains were isolated from the Klíčava and Římov reservoirs. Their cells are rather small, cocci to short rods, with the volume 0.03–0.05 µm^3^. The existence of the LimB lineage has been indicated previously by clone PRD01b009B (AF289169) related sequences retrieved from Lake Michigan where it constituted the highest proportion of clones of freshwater *Betaproteobacteria* [30].

**Lineage LimC** includes two described species *L. planktonicus* and *L. parvus*, 25 newly isolated strains presented in this study (Table 2) and other environmental sequences. The origin of the sequences affiliated within the LimC lineage is worldwide (e.g. Europe, USA, Argentina, Taiwan and China) including not only free-living genotypes from freshwater habitats and estuaries but also genotypes described from epithelium of *Hydra vulgaris* [31] and digestive tract of *Daphnia magna* [32]. This lineage harbors all the bacterial morphotypes found, i.e. cocci, rods and solenoid bacteria (cf Fig. 1). The affiliated strains share similarities in both their 16S rRNA genes (>98.4%) and their IGS1 sequences (>89%). We propose the following annotation and differentiation, indicated by morphologically similar genetic clusters (Fig. 4). All proposed sublineages have been consistently observed in trees, however their phylogenetic position within the lineage LimC in not fully supported by bootstrap analyses. **LimC1** and **LimC2 sublineages** are proposed for species clusters of *L. planktonicus* and *L. parvus,* respectively. The morphological and genetic similarities of strains 2KL-16 (Fig. 1J) and Rim42 with *L. planktonicus* II-D5^T^ suggest that they probably represent the same species. Strains LI2-55 (Fig. 1H) and VIII-A6 possess identical IGS1 and 16S rRNA gene sequences as strain *L. parvus* II-B4^T^ and similar morphology, thus they likely represent the same species. However, strain LI2-55 was isolated from a habitat located 700 km far from the habitat of VIII-A6 and II-B4^T^. The **sublineage LimC3** harbors two coccoid strains CEP5 (Fig. 1F) and T6-20 isolated from habitats with high nutrient concentration. The **sublineage LimC4** is proposed for strains 15K, Rim47, B22-3k and Hin4 (Fig. 1I), representing short rods/cocci. Also sequences gained from the epithelium of *Hydra* sp. [31] are most likely affiliated within this lineage. The strains were recovered from different types of habitats, an eutrophic pond, a mesotrophic reservoir and a calcareous alpine lake. The **sublineage LimC5** contains coccoid morphotypes of bacteria and is represented by strains 2KL-27 (Fig. 1G) and KL5. Both strains were isolated from one mesotrophic reservoir however originating from different samplings (Fig. 4). The morphologically exceptional **sublineage LimC6** (cf. Fig. 1L) is composed of strains 2KL-3, 2KL-7, G3-3, SP2 and WS1. They are characterized by largest cell volumes (up to 1 µm^3^) found within *Limnohabitans* genus so far, as well as by their clearly distinguishable C-shaped morphology. Interestingly, sequences obtained from the digestive tract of *Daphnia magna* [32] are affiliated within this sublineage. Its members were generally indigenous dwellers of lacustrine environments, a mesotrophic reservoir and eutrophic shallow ponds.

The existence of **LimD lineage** is highly supported by bootstrap analysis (Fig. 4), however, it still does not include any isolated strain and is defined exclusively on the basis of the corresponding environmental sequences obtained from Genbank. In the previous study [3], sequences of this group have been associated with Lhab-A2 lineage and synonymized with the GKS16 cluster defined by Zwart et al. [9] closely related to the *Polaromonas* genus. To resolve the phylogenetic position of the lineage Lhab-A2, we added our strains and members of genera *Curvibacter, Rhodoferax* and *Polaromonas* into the Newton’s ARB database [3] of environmental clones and we reconstructed the alignment and recalculated phylogenetic trees from partial 16S rRNA gene sequences. Surprisingly, the results differed from previous analyses lacking ARB data (including IGS1 sequences analyses). Similarly, the results differed when different lengths of sequences were used. We could solve the problem only by modifying ARB alignment for all *Betaproteobacteria* with the help of helices predictions in OligoAnalyzer 3.1 (Integrated DNA Technologies, Inc) or with alignment from Mafft [33]. Our analyses show that Lhab-A2 tribe (as defined by Newton et al. [3]) is composed of two phylogenetically unrelated lineages – one containing both the R-BT065 and Rho-BAL47 determinative cluster sequences (e.g. EU803573 or AF534429), another lacking both and being related to the GKS16 clone and to the *Polaromonas* genus (e.g. EU640680 or FJ849147). For future purposes, we propose to delineate the **LimD** lineage of the *Limnohabitans* genus, with the clones FukuN55 (AJ289999) and PIB-25 (AM849436) as “type” sequences, from the **GKS16** lineage related to the *Polaromonas* genus, containing clones GKS16 (AJ224987) and JEG.e1 (DQ228403). The LimD sequences clustering within this lineage originate from oligo- to mesotrophic lakes in Austria, Germany and Switzerland [5], [18], [34], [35] as well as from estuary of Delaware river [36]. In contrast, the sequences affiliated within the GKS16 lineage were retrieved almost exclusively from cold habitats (i.e. snow, ice core, arctic streams) whereas no *Limnohabitans* sequences have been obtained from such habitats to date. However, there is an evidence that both lineages can co-occur in the same habitat, e.g. high mountain lakes [11], [24].

**LimE lineage** consists only of two strains isolated from the same habitat, however, morphologically diversified (Table 2). Its members are genetically close to the lineage LimA, but they can be hybridized with R-BT065 probe (Fig. 3 and Fig. 4). This lineage probably includes the “R-BT065” subcluster indicated in Newton’s ARB database, represented by 58 clones described exclusively from the Lake Michigan [30], e.g. clones LW1m-1-53 (EU639913) and GC1m-1-33 (EU641261). However, this lineage requires a revision when more IGS sequences will be obtained.

### Biovolume of R-BTs

**Figure 5:**
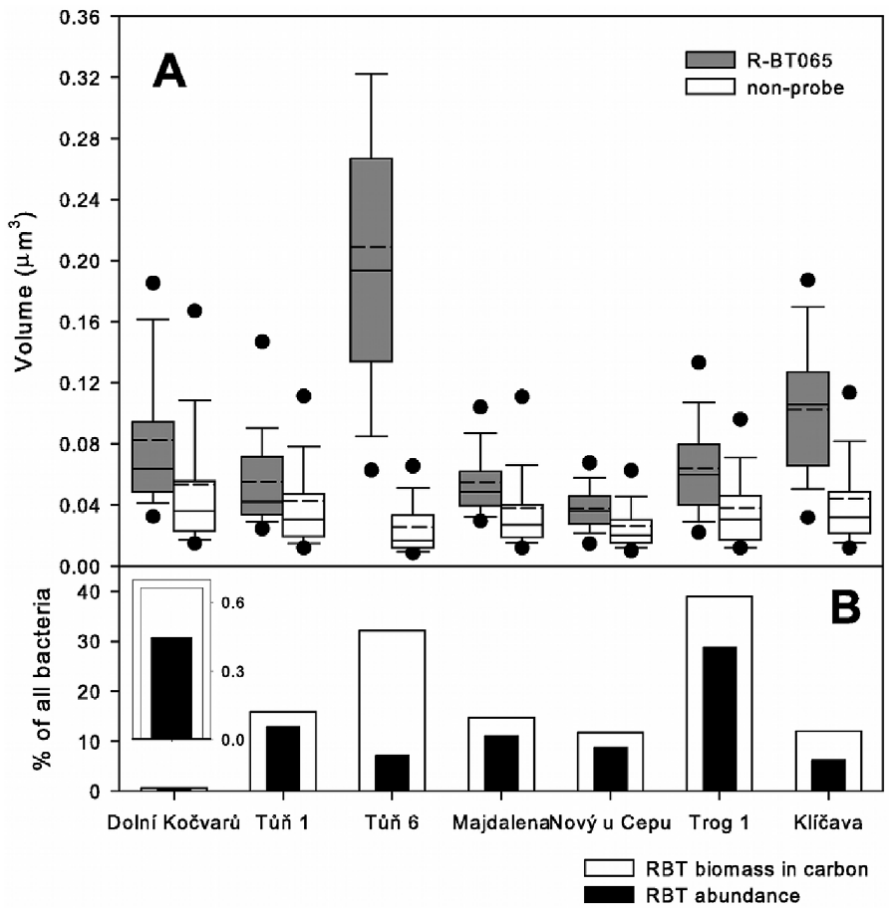
Biovolume of the R-BT065-positive-cells compared to other bacteria and their relative contribution in natural bacterial community. (A) Boxes represent 25% and 75% quartils, whiskers 5% and 95% quintiles, full circles outliers. Dashed lines represent means, whereas full lines are medians. (B) Relative proportions (%) of the R-BT065 bacteria targeted to total cells (black bar) and to total carbon biomass (white bar). Note that due to very low proportion of the R-BT bacteria in humic pond Dolní Kočvarů there is also incorporated a fine-scale resolution insert.

Volumes of all heterotrophic and all R-BT bacteria (targeted with R-BT065 probe) were determined for 7 different habitats that were selected on the basis of our previous knowledge on R-BT bacteria abundance (Fig. 5). Volume of R-BT cells ranged from 0.003 to 0.685 µm^3^ whereas the volume of non-R-BT bacteria ranged from 0.003 to 0.224 µm^3^ for all habitats. The R-BT cells possessed significantly higher MCV (mean cell volume) in “Tůň 6” pond (0.209 µm^3^) as compared to other habitats. This habitat was dominated by curved rod cells similar to T6-5 strain (0.3–0.9 µm^3^) which has been isolated from. The habitat was characterized by a bloom of oiled chrysophytes in neuston, an oxygen depletion (1.7 mg l^−1^ O_2_), and unusually high phosphate (771.1 µg l^−1^ DRP) and ammonium nitrogen (1.04 mg l^−1^ NH_4_-N) concentrations during the time of sampling. Surprisingly, the volumes of non-R-BT cells didn’t differ from those in other habitats. Second highest R-BT cell volumes (MCV of 0.103 µm^3^) were detected in Klíčava reservoir which serves for drinking water supply [11]. On the other hand, the lowest average values were found in “Nový u Cepu” pond, Majdalena sand pit and “Tůň 1” pond (0.038, 0.055 and 0.056 µm^3^, respectively). In all examined habitats, MCVs of R-BT cells were consistently larger than those of the non-R-BT cells (p<0.001, Fig. 5A). Thus the relative contribution of R-BTs to total bacterial biomass in the cellular carbon was in all cases significantly larger than their relative abundance (Z = 2.366, p = 0.016, Fig. 5B).

## Discussion

### Betaproteobacteria – ecological Relevance versus Available Isolates

One of the fundamental goals of the field freshwater microbial ecology is connecting our rather limited knowledge on the “not-easily cultivable” but key bacterioplankton taxa with their major environmental functions [3]. Due to the inherent difficulty in the cultivability of aquatic bacteria (e.g. [9], [37], the mosaic of the relevant taxonomic units and especially their function remains largely incomplete. In this study, we present a first overview of the morphological, genetic and physiological microdiversity within the *Limnohabitans* genus based on newly isolated strains with a large potential to link data on genetic diversity to data on phenotypic diversity and ecological roles of particular taxonomic units.

Freshwater *Betaproteobacteria* represent a group of heterotrophic bacteria with the largest number of so far isolated strains, although most of them belong to the *Polynucleobacter* genus [38], [39], [40], [41]. Our study reports on 35 newly isolated strains from the *Limnohabitans* genus [4] an important unit of the BetI clade [9]. Notably, another 16 *Limnohabitans* strains were recently isolated from lakes Teganuma, Inbanuma, Inawashiro and Ushikunuma on Japan islands (K. Watanabe et al., unpublished results). Thus, including four described species there are currently at least 55 distinct strains available for further studies.

### Revision of the Phylogenetic Scheme for Freshwater Comamonadaceae

Hundreds of partial 16S rRNA gene sequences in Genbank (www.ncbi.nih.gov) retrieved by cultivation-independent approaches and affiliated within the R-BT lineage and/or the genus *Limnohabitans* give the potential of a plausible phylogenetic reconstruction of the genus [3], [7], (Fig. 3) in this study. Our newly isolated strains are affiliated within the *Limnohabitans* genus with high similarities of their 16S rRNA gene sequences (Table S1 in File S1). Phylogenetic analysis of 16S rRNA genes revealed five main lineages within the genus (Fig. 3). Two of them, which contain already described species, are in concordance with Lhab-A1 and Lhab-A3 clades proposed by Newton and coworkers [3]. However, some of our phylogenetic reconstructions contradict the proposals presented in the later paper by Newton et al. Contrasting results are probably a consequence of low discriminative value of 16S rRNA gene sequence in the BetI lineage which coincide with single base variability within genera *Limnohabitans* and *Polaromonas*. We recommend to circumvent such an ambiguity by using of another genetical markers, i.e. IGS1 sequence.

The affiliation of the clade Lhab-A4 within the *Limnohabitans* genus is highly questionable. The phylogenetic analyses suggest the position of Lhab-A4 as a sister lineage of the *Limnohabitans* genus or at the edge of this genus. Moreover, none of the Lhab-A4 clones, e.g. clones ADK-MOe02-95 (EF520475) and LW9m-3-24 (EU641662) contain the target sequence for the R-BT065 probe, however they could be targeted with the Rho-BAL47 probe [9]. Nevertheless, the lack of isolated members does not allow to tell, whether lineage Lhab-A4 could be assigned to the *Limnohabitans* genus or not. We intend to leave the question open until additional markers are available.

In contrast to the previously proposed phylogenetic scheme [3], there is compelling evidence for the existence of five *Limnohabitans* lineages (or six when Lhab-A4 is considered): four lineages representing the R-BT bacteria and one lineage (LimA) for non-R-BT bacteria (Figs. 3 and 4). Based on the resolution of our phylogenetic analysis on existing isolated strains, we propose a new phylogenetic scheme for the Beta-I group and new names for the respective lineages within the *Limnohabitans* genus (Fig. 3), which substantially refines and clarifies the scheme suggested by Newton et al. [3]. Unfortunately, the 16S rRNA sequence nucleotide composition does not permit to design FISH probes specific to individual lineages to be detected in environmental samples.

### Fine-scale Resolution within the Genus

The availability of a broad spectrum of strains from the same lineage allows testing the suitability of markers for a finer resolution at the species-level in natural habitats. An important contribution of our research is the sequencing of the highly variable 16-23S rRNA intergenic spacer (IGS1). To the best of our knowledge, IGS1 sequences of uncultured or cultivated *Limnohabitans* strains were not previously deposited in Genbank. An explanation of the widespread avoidance of IGS1 sequencing is the possible presence of multiple operons of the ribosomal genes and the presence of the multiple non-identical IGS1 sequences in a single genome [42]. However, recently published draft genomes of two *Limnohabitans* strains contain only single copies of all ribosomal genes clustered in one complete rRNA operon [28]. Moreover, only two rRNA operons, but with identical IGS1 sequences, were reported in closely related *Rhodoferax ferrireducens* genome [43], and only one rRNA operon seems to be present in a common freshwater betaproteobacterium *Polynucleobacter necessarius* spp. *asymbioticus* genome [41]. Moreover, the highest intragenomic divergence of IGS1 sequences within *Betaproteobacteria* was reported being about 5% [44], while we found a IGS1 sequence similarity higher than 89% within proposed lineages (Table S2 in File S1).

IGS1 sequences have been frequently used to distinguish closely related strains [38], [45], [46], [47]. Therefore, six genotype groups (LimC1-C6), including two to four strains with similar size and shape as well as identical IGS1 and 16S rRNA gene sequences and isolated from more than one habitat, were explicitly proposed as new well-defined taxonomic units (c.f. Figs. 3 and 4). Regarding the morphological features of the isolated strains, we hypothesize that the lower limit of the IGS1 similarity within an individual genotype is about 95% (Table S2 in File S1), which permits consideration of all other strains as genotypes as-well. However, the similarity of genes and the similarity of the whole bacterial genomes do not correlate [48]. Our data suggest (Fig. 2), that there are at least eight (II-D5 vs. 2KL-16) or six (B22-3k vs. 15K) metabolic differences between the strains clustered within the proposed phylogenetic sublineages LimC1 and LimC4, respectively. Moreover, it seems that strains with a broad substrate spectrum (aka opportunist) are affiliated together with specialists (e.g. in LimC4 sublineage). Thus, additional isolation, phylogenetic analyses of multiple genes and physiologic tests are needed to verify our hypothesis since further splitting of the proposed sublineages (or groups) could not be ruled out.

Contrary to our expectations, it seems impossible to draw firm conclusions on habitat preferences of proposed *Limnohabitans* (sub-)lineages based solely on 16S rRNA sequences deposited in Genbank (Table S1 in File S1). Several reasons could be hypothesized: (i) We have too rough phylogenetic resolution, thus the ecological diversification of these bacteria is undoubtedly deeper than currently mirrored by available molecular data [47]. (ii) We have only limited knowledge on the ecology of this bacterial group and we are still missing essential drivers of ecological diversification.

### Are there Common Traits among Limnohabitans Members?

The ability to respond to changing conditions, called “metabolic IQ” [49], has been suggested to be correlated with the bacterial genome size and in turn also with their cell volume [50]. If these assumptions are correct, the generally larger cell volume (Fig. 5) and the growth potential of the R-BT bacteria [20] indicate that they belong to the opportunistic (i.e. more substrate-responsive) fraction of the bacterioplankton (c.f. [18]). Such a hypothesis is supported by our data. All strains, tested in this work, share the ability to increase their biomass on simple organic acids and sugars and most of them were able to use more than one substrate (Fig. 2). Moreover, two draft genomes of *Limnohabitans* strains Rim28 and Rim47 revealed a potential for photosynthesis, CO_2_ fixation, ammonia- and sulfur-oxidation and a genome size around 3.2 Mb with about 3000 of ORFs [28]. Thus, a great metabolic versatility could be expected in the *Limnohabitans* genus and its members seem to belong to *Betaproteobacteria* with appreciable “metabolic IQ”.

Environmental factors such as pH, conductivity, and the proportion of low-molecular-weight compounds in dissolved organic carbon were found to correlate with their abundance throughout a large spectrum of lakes [11]. The 16S rRNA gene libraries contain only few clone sequences affiliated with the *Limnohabitans* genus, described from acidic habitats, i.e. Adirondack lakes [35]. However, the clones were also indigenous to the Cascade Lake, e.g. ADK-CSe02-53 (EF520468), or to the Moss Lake, e.g. ADK-MOe02-95 (EF520475) characterized with pH >6. Surprisingly, no significant correlation of the abundance of R-BT bacteria with lake trophic status and chlorophyll a concentration was found [11], nor any clear habitat preference can be determined for individual lineages from the phylogenetic distribution of sequences deposited in Genbank (Table S3 in File S1). Slightly different results were recently described for clades Lhab-A2 and Lhab-A4 from an annual dynamic of the Lake Erken [29]. Both clades were negatively linked to temperature and total bacterial abundance. Moreover, clade Lhab-A4 was negatively link to pH, chlorophyll a, particulate nitrogen and phosphorus.

### Large Potential for Ecological Differentiation

The success in isolation of strains possessing frequently different ecophysiology from the same habitat or even from the same water sample (Table 1 and 2) and the existence of clone libraries with sequences distributed throughout all *Limnohabitans* lineages, e.g. [36], suggest that their coexistence is likely facilitated by their different ecophysiological traits. In addition, the high abundance of *Limnohabitans* members (in average 0.3 10^6^ ml^−1^) [11] together with large genetic diversity (c.f. DNA-DNA hybridization values in [4], [6] and [7]) indicate a huge potential for diversification and speciation.

Three putative mechanisms for the speciation and niche differentiation within the same body of water can be proposed based on physiological traits of isolated strains and available knowledge on the R-BT lineage ecology.

Metabolic capabilities of the bacteria are assumed to give them a specific physiological potential to exploit available organic carbon. This potential is variable for individual *Limnohabitans* strains (Fig. 2). Despite our database is incomplete, most of strains showed marked differences in substrate utilization and we hypothesize that each of them inhabits its own specific niche. The quality of the organic matter is not only coupled to its allochthonous and autochthonous origin (e.g. [24]), but even to particular algal or cyanobacterial producers (e.g. [51], [52]). The changes in bacterial community composition, and species-specific algal-bacterial relationships have been documented in both marine and freshwaters [53], [54], [55]. Moreover, the algal-specific coupling was described for R-BT bacteria [25], [26], [54]. The investigations on the potential of two tested *Limnohabitans* species to use algal derived organic matter showed significant differences in their growth characteristics [26].

The morphological and size-related diversity present within the R-BT bacteria (see Figs. 1 and 5) likely corresponds also with a different degree of their vulnerability to grazing. This is supported by investigations of the ecological traits of two closely related, but in size and morphology rather dissimilar bacteria, i.e. *L. planktonicus* and *L. parvus* [23]. Strain-specific differences in the vulnerability to flagellate grazing and to viral infection [23] suggest that these species occupy separated ecological niches [56]. The cell volume of the newly isolated strains encompass a range from 0.03 up to 1 µm^3^ (Table 2), thus according to marine bacteria their genome size could range from about 1.6 to 6 Mbp [50]. Although these approximations are only rough and might be incorrect, there is a certain possibility that at least some small cell-sized R-BT bacteria could harbor small-sized genomes with a low metabolic potential. Then for escaping grazing pressure they could exploit the so-called “cryptic escape” lifestyle suggested by Yooseph et al. [50] instead of the above mentioned opportunist strategy with high metabolic IQ.

Finally, the presence of members of the *Limnohabitans* genus have been reported by non-cultivable methods from exotic aquatic sites: the epithelium of free-living *Hydra* [31], and the gut microflora of *Daphnia magna* [32], cf. Fig. 3). It seems that such a possible symbiosis or mutualism might be more common for distinct aquatic bacterial genera. Similar types of associations were described for the freshwater genus *Polynucleobacter* [57] or the marine genus *Vibrio* [58]. These associations are highly (strain) specific and the bacterial symbiont occupies a privileged niche, which highly modifies its life strategy in an aquatic habitat.

### Concluding Remarks

Previously an uncultured bacterial group now contain a large number of distinct members. We can assume that there is enough information to open a black box frequently used in the research on freshwater microbial ecology (for review see [3]) and assign the target group of bacteria to new phylogenetically defined taxa with distinct phenotypic and ecological features. To determine the well-defined ecological units of the *Limnohabitans* genus, it is of the primary interest to study the biological interactions on the species- or even strain-level. In addition, there is an urgent need to establish narrower, high taxonomic-resolution markers to describe the occurrence, habitat preferences and ecological roles of individual *Limnohabitans* lineages and genotypes. We propose the IGS1 sequence as a more appropriate marker than the commonly used 16S rRNA gene for fine-scale phylogenetic studies within the *Limnohabitans* genus, and we provide a basic sequence dataset and a taxonomic framework both suitable for interpretation of clone libraries established by cultivation-independent methods.

## Experimental Procedures

### Isolation and Identification of Bacteria

Bacterial strains were isolated from freshwater reservoirs, lakes and ponds in the Czech Republic, Austria, the Netherlands and France (Corse) using a modified protocol of the acclimatization method [37]; for more details of the habitats used for isolation, see Table 1 and [23]. We state that no specific permissions were required for sampling of any locations and that locations were not privately-owned or protected in any way. We confirm that the field studies did not involve endangered or protected species. Two manipulation approaches were used to enrich bacteria affiliated with the *Limnohabitans* genus, either a grazer removal or a sample dilution approach. The first protocol, as described by Kasalický et al. [7], employed the filtration of the whole water sample through 0.8 µm polycarbonate membrane filter (OSMONICS, Livermore, USA) to remove protists. In the second method, the whole water sample was diluted 1∶1 with Inorganic Basal Medium (IBM, [37]. After both manipulations, the samples were kept for 24 hours in dark, facilitating enhanced bacterial growth and activation, and subsequently diluted with sterile IBM medium in order to obtain cell concentrations suitable for inoculation of 24-well microplates with approximately 0.5 cells per well. Usually 6 to 10 microplates were used for one water sample. The established cultures were stepwise acclimatized by additions of increasing doses of NSY medium to growth at 3 g l^−1^ [37]. Wells showing turbidity were screened by FISH with the Bet42a (whole *Betaproteobacteria*, [59] and the R-BT065 (R-BT lineage [8]) probes for presence of *Limnohabitans* spp. Samples were scored as “positive” when the cells hybridized with the R-BT065 probe or solenoids hybridized only with Bet42a probe (for the strains related to *L. curvus* and *L. australis* (c.f. [4], [6]). 500 µl from the positive wells were re-inoculated to fresh NSY medium and at least 3 times purified by dilution to extinction. The purity of cultures was controlled microscopically by DAPI staining, by FISH [8], and by growth on agar plates (NSY medium). However, not all cultures were able to grow on solid media (1.5% agar), thus the latter test provided only partial or additional information on the purity of a culture based on colony size, shape and color.

### Metabolic Tests

The isolated strains were routinely grown in liquid NSY medium with strength of 3 g l^−1^. Assimilation experiments were performed by comparison of optical density measured at 575 nm (OD_575_) established in liquid one-tenth-strength NSY medium (0.3 g l^−1^) with and without 0.5 g of a test substance per liter (pH 7.2), as described previously [39]. Differences in OD_575_ were scored as ++(highly positive, >180% of control treatments), +(positive, 50–180%), w (weak, 10–50%), − (no growth, −20–10%), – (light inhibition, −40–−20%) and inhib (severe inhibition, <−40%).

### Phylogenetic Analysis

DNA from the established purified cultures was extracted by using the UltraClean™ isolation kit (MoBio, Laboratories, Inc.). The 16S rRNA genes and the intergenic spacer region between 16S and 23S rRNA genes (IGS1) were amplified using primers 27F, 1492r (both [60]), and 1406F [61], 23Sr [62] as described in Hahn et al. [38]. The PCR products were purified by Nucleospin™ (MoBio, Laboratories, Inc.). Sequencing was performed commercially by Eurofins MWG Operon (Germany). To obtain IGS1 sequences of closely-related reference species, the following strains were grown in 3 g.l^−1^ NSY medium: *L. australis* MWH-BRAZ-DAM2D^T^, *L. curvus* MWH-C5^T^, *L. parvus* II-B4^T^, *L. planktonicus* II-D5^T^, *Curvibacter gracilis* 7-1^T^ and *Rhodoferax fermentans* FR2^T^.

Sequences were aligned with MAFFT 6 (http://mafft.cbrc.jp/alignment/server) [33], [63]. Aligned sequences were edited in BioEdit 7.0 [64]. Similarities of aligned sequences were calculated by the Sequence Identity Matrix program in BioEdit 7.0, and pairwise distances were calculated with MEGA 5 [65]. Best model for Maximum Likelihood (GTR+Γ+I) analysis was estimated by jModelTest [66]. Neighbor-joining trees and Maximum Parsimony were calculated by using the software MEGA 5 [65], Maximum Likelihood trees were generated using PhyML 3.0 [67], Bayesian evolution was calculated by using MrBayes 3.1.2 [68]. Additional phylogenetic analyses with the Newton’s dataset of environmental clones enlarged by new sequences were conducted in ARB [69]. The internal alignment program and ARB Neighbor Joining algorithm were used for phylogenetic analyses.

### Biovolume of the FISH-positive Bacteria in Natural Samples

Natural samples (10 to 20 ml) for catalyzed reporter deposition FISH were sampled as described in [23]. Cells were hybridized with the R-BT065 oligonucleotide probe [8]. The proportions of FISH-positive bacteria were determined directly by inspecting 600 to 1,000 cells in replicated samples using epifluorescence microscopy (Olympus AX-70). Gray-scale images of bacterial cells were acquired with a CCD camera in two channels with distinct combination of excitation and emission light spectra. The “probe” channel was used to assign the R-BT065-positive cells to their image in “DAPI” channel. Cell sizing, based on measuring of cell width and length, was conducted in “DAPI” channel by using the semiautomatic image analysis system LUCIA D (Lucia 3.52; Laboratory Imaging, Prague, Czech Republic) as described by [70] and [71]. Between 30 and 100 hybridized cells were measured per sample to determine the mean cell volume (MCV) of the R-BT065-positive bacteria. Cell volumes of probe detected and not-targeted bacteria were compared by Mann-Whitney U statistical test, since the normality distribution test failed (p<0.001).

Carbon content of individual cells was calculated according to Loferer-Krössbacher et al. [72]. The relative proportions of abundance and carbon biomass of R-BT065-positive cells in selected habitats were calculated using the cluster-specific abundance given in Šimek et al. [23] and were compared to the values for all bacterioplankton cells by Wilcoxon Signed Rank Test (pair t-test for data where normality test failed, p = 0.020).

### Nucleotide Sequences

16S rRNA gene sequences and 16S–23S IGS1 sequences of the *Limnohabitans* isolates and several reference strains were deposited under the Accession Numbers HE600660–HE600692. A detailed list of strains and the corresponding accession numbers is available in Table S3 in File S1.

## Supporting Information

File S1 **Table S1.** Pairwise comparisons of aligned almost complete 16S rRNA gene sequences of newly isolated *Limnohabitans* strains and closely related environmental clones and other genera. The similarity is shown in the upper part, the lower part depicts the number of nucleotide differences between sequences. Newly described strains are in bold. Environmental sequences are mostly represented by their accession number. See Table S3 for their labels and details. Similar sequences and sequences with max 1 mismatch are depicted in green. **Table S2.** Pairwise comparisons of complete 16S–23S rRNA intergenic spacer (IGS1) sequences of *Limnohabitans* strains and closely related species. The similarity is shown in the upper part, the lower part depicts the number of mismatches between sequences. Sequences that are similar >97% or their difference is not higher than 24 nucleotides are depicted in green. **Table S3.** Accession numbers of sequences from bacterial strains and environmental clones used in this work.
(ZIP)

## References

[1] Glöckner FO, Fuchs BM, Amann R (1999) Bacterioplankton compositions of lakes and oceans: a first comparison based on fluorescence in situ hybridization. Appl Environ Microbiol 65: 3721–3726. 10427073 10.1128/aem.65.8.3721-3726.1999 PMC91558

[2] Lindström ES, Kamst-Van Agterveld MP, Zwart G (2005) Distribution of typical freshwater bacterial groups is associated with pH, temperature, and lake water retention time. Appl Environ Microbiol 71: 8201–8206. 16332803 10.1128/AEM.71.12.8201-8206.2005 PMC1317352

[3] Newton RJ, Jones SE, Eiler A, McMahon KD, Bertilsson S (2011) A guide to the natural history of freshwater lake bacteria. Microbiol Mol Biol Rev 75: 14–49. 21372319 10.1128/MMBR.00028-10 PMC3063352

[4] Hahn MW, Kasalický V, Jezbera J, Brandt U, Jezberová J, et al (2010a) *Limnohabitans curvus* gen. nov, sp. nov, planktonic bacterium isolated from a freshwater lake. Int J Syst Evol Microbiol 60: 1358–1365. 19671731 10.1099/ijs.0.013292-0 PMC3091418

[5] Glöckner FO, Zaichikov E, Belkova N, Denissova L, Pernthaler J, et al (2000) Comparative 16S rRNA analysis of lake bacterioplankton reveals globally distributed phylogenetic clusters including an abundant group of *Actinobacteria* . Appl Environ Microb 66: 5053–5065. 10.1128/aem.66.11.5053-5065.2000 PMC92419 11055963

[6] Hahn MW, Kasalický V, Jezbera J, Brandt U, Šimek K (2010) *Limnohabitans australis* sp. nov, isolated from a freshwater pond, and emended description of the genus *Limnohabitans.* . Int J Syst Evol Microbiol 60: 2946–2950. 20118294 10.1099/ijs.0.022384-0 PMC3031073

[7] Kasalický V, Jezbera J, Šimek K, Hahn MW (2010) *Limnohabitans planktonicus* sp. nov. and *Limnohabitans parvus* sp. nov, planktonic betaproteobacteria isolated from a freshwater reservoir, and emended description of the genus *Limnohabitans* . Int J Syst Evol Microbiol 60: 2710–2714. 20061501 10.1099/ijs.0.018952-0 PMC3091486

[8] Šimek K, Pernthaler J, Weinbauer MG, Horňák K, Dolan JR, et al (2001) Changes in bacterial community composition, dynamics and viral mortality rates associated with enhanced flagellate grazing in a meso-eutrophic reservoir. Appl Environ Microbiol 67: 2723–2733. 11375187 10.1128/AEM.67.6.2723-2733.2001 PMC92931

[9] Zwart G, Crump BC, Kamst-van Agterveld MP, Hagen F, Han SK (2002) Typical freshwater bacteria: an analysis of available 16S rRNA gene sequences from plankton of lakes and rivers. Aquat Microb Ecol 28: 141–155.

[10] Page KA, Connon SA, Giovannoni SJ (2004) Representative freshwater bacterioplankton isolated from Crater Lake, Oregon. Appl Environ Microbiol 70: 6542–6550. 15528517 10.1128/AEM.70.11.6542-6550.2004 PMC525233

[11] Šimek K, Kasalický V, Jezbera J, Jezberová J, Hejzlar J, et al (2010) Broad habitat range of the phylogenetically narrow R-BT065 cluster representing a core group of the betaproteobacterial genus *Limnohabitans* . Appl Environ Microbiol 76: 631–639. 19948856 10.1128/AEM.02203-09 PMC2812994

[12] Hörtnagl P, Pérez MT, Zeder M, Sommaruga R (2010) The bacterial community composition of the surface microlayer in a high mountain lake. FEMS Microbiol Ecol 73: 458–467. 20528985 10.1111/j.1574-6941.2010.00904.x PMC2955963

[13] Buck U, Grossart HP, Amann R, Pernthaler J (2009) Substrate incorporation patterns of bacterioplankton populations in stratified and mixed waters of a humic lake. Environ Microbiol 11: 1854–1865. 19320716 10.1111/j.1462-2920.2009.01910.x

[14] Salcher MM, Pernthaler J, Posch T (2010) Spatiotemporal distribution and activity patterns of bacteria from three phylogenetic groups in an oligomesotrophic lake. Limnol Oceanogr 55: 846–856.

[15] Šimek K, Horňák K, Jezbera J, Mašín M, Nedoma J, et al (2005) Influence of top-down and bottom-up manipulations on the R-BT065 subcluster of b-proteobacteria, an abundant group in bacterioplankton of a freshwater reservoir. Appl Environ Microbiol 71: 2381–2390. 15870325 10.1128/AEM.71.5.2381-2390.2005 PMC1087523

[16] Salcher MM, Hofer J, Horňák K, Jezbera J, Sonntag B, et al (2007) Modulation of microbial predator-prey dynamics by phosphorus availability: growth patterns and survival strategies of bacterial phylogenetic clades. FEMS Microbiol Ecol 60: 40–50. 17250752 10.1111/j.1574-6941.2006.00274.x

[17] Horňák K, Jezbera J, Nedoma J, Gasol JM, Šimek K (2006) Effects of resource availability and bacterivory on leucine incorporation in different groups of freshwater bacterioplankton, assessed using microautoradiography. Aquat Microb Ecol 45: 277–289.

[18] Salcher MM, Pernthaler J, Zeder M, Psenner R, Posch T (2008) Spatio-temporal niche separation of planktonic *Betaproteobacteria* in an oligo-mesotrophic lake. Environ Microbiol 10: 2074–2086. 18430016 10.1111/j.1462-2920.2008.01628.x

[19] Pérez MT, Hörtnagl P, Sommaruga R (2010) Contrasting ability to take up leucine and thymidine among freshwater bacterial groups: implications for bacterial production measurements. Environ Microbiol 12: 74–82. 19725866 10.1111/j.1462-2920.2009.02043.x PMC2810431

[20] Šimek K, Horňák K, Jezbera J, Nedoma J, Vrba J, et al (2006) Maximum growth rates and possible life strategies of different bacterioplankton groups in relation to phosphorus availability in a freshwater reservoir. Environ Microbiol 8: 1613–1624. 16913921 10.1111/j.1462-2920.2006.01053.x

[21] Šimek K, Weinbauer MG, Horňák K, Jezbera J, Nedoma J, et al (2007) Grazer and virus-induced mortality of bacterioplankton accelerates development of *Flectobacillus* populations in a freshwater community. Environ Microbiol 9: 789–800. 17298377 10.1111/j.1462-2920.2006.01201.x

[22] Jezbera J, Horňák K, Šimek K (2006) Prey selectivity of bacterivorous protists in different size fractions of reservoir water amended with nutrients. Environ Microbiol 8: 1330–1339. 16872397 10.1111/j.1462-2920.2006.01026.x

[23] Šimek K, Kasalický V, Horňák K, Hahn MW, Weinbauer MG (2010) Assessing niche separation in coexisting *Limnohabitans* strains through interactions with a competitor, viruses, and a bacterivore. Appl Environ Microbiol 76: 1406–1416. 20038688 10.1128/AEM.02517-09 PMC2832377

[24] Pérez MT, Sommaruga R (2006) Differential effect of algal- and soil-derived dissolved organic matter on alpine lake bacterial community composition and activity. Limnol Oceanogr 51: 2527–2537.

[25] Šimek K, Horňák K, Jezbera J, Nedoma J, Znachor P, et al (2008) Spatio-temporal patterns of bacterioplankton production and community composition related to phytoplankton composition and protistan bacterivory in a dam reservoir. Aquat Microb Ecol 51: 249–262.

[26] Šimek K, Kasalický V, Zapomělová E, Horňák K (2011) Algal-derived substrates select for distinct betaproteobacterial lineages and contribute to niche separation in *Limnohabitans* strains. Appl Environ Microbiol 77: 7307–7315. 21873481 10.1128/AEM.05107-11 PMC3194872

[27] Glaeser SP, Grossart H-P, Glaeser J (2010) Singlet oxygen, a neglected but important environmental factor: Short-term and long-term effects on bacterioplankton composition in a humic lake. Environ Microbiol 12: 3124–3136. 20636377 10.1111/j.1462-2920.2010.02285.x

[28] Zeng Y, Kasalický V, Šimek K, Koblížek M (2012) Genome sequences of two freshwater betaproteobacterial isolates, *Limnohabitans* species strains Rim28 and Rim47, indicate their capabilities as both photoautotrophs and ammonia oxidizers. J Bacteriol 194 (22): 6302 doi: 10.1128/JB.01481–12 10.1128/JB.01481-12 PMC3486343 23105051

[29] Eiler A, Heinrich F, Bertilsson S (2012) Coherent dynamics and association networks among lake bacterioplankton taxa. ISME J 6: 330–342. 21881616 10.1038/ismej.2011.113 PMC3260505

[30] Mueller-Spitz SR, Goetz GW, McLellan SL (2009) Temporal and spatial variability in nearshore bacterioplankton communities of Lake Michigan. FEMS Microbiol Ecol 67: 511–522. 19220863 10.1111/j.1574-6941.2008.00639.x

[31] Fraune S, Bosch T (2007) Long-term maintenance of species-specific bacterial microbiota in the basal metazoan *Hydra*. Proc Natl Acad Sci U S A, 104, 13146–13151. 10.1073/pnas.0703375104 PMC1934924 17664430

[32] Freese HM, Schink B (2011) Composition and stability of the microbial community inside the digestive tract of the aquatic crustacean *Daphnia magna*. Microb Ecol DOI 10.1007/s00248–011–9886–8. 10.1007/s00248-011-9886-8 21667195

[33] Katoh K, Toh H (2008) Recent developments in the MAFFT multiple sequence alignment program. Brief Bioinform 9: 286–298. 18372315 10.1093/bib/bbn013

[34] Crump B, Kling G, Bahr M, Hobbie J (2003) Bacterioplankton community shifts in an Arctic lake correlate with seasonal changes in organic matter source. Appl Environ Microbiol 69: 2253–2268. 12676708 10.1128/AEM.69.4.2253-2268.2003 PMC154827

[35] Percent SF, Frischer ME, Vescio PA, Duffy EB, Milano V, et al (2008) Bacterial community structure of acid-impacted lakes: what controls diversity? Appl Environ Microbiol 74: 1856–1868. 18245245 10.1128/AEM.01719-07 PMC2268290

[36] Shaw AK, Halpern AL, Beeson K, Tran B, Venter JC, et al (2008) It’s all relative: ranking the diversity of aquatic bacterial communities. Environ Microbiol 10: 2200–2210. 18637951 10.1111/j.1462-2920.2008.01626.x

[37] Hahn MW, Stadler P, Wu QL, Pöckl M (2004) The filtration-acclimatization-method for isolation of an important fraction of the not readily cultivable bacteria. J Microb Meth 57: 379–390. 10.1016/j.mimet.2004.02.004 15134885

[38] Hahn MW, Pöckl M, Wu QL (2005) Low intraspecific diversity in a *Polynucleobacter* subcluster population numerically dominating bacterioplankton of a freshwater pond. Appl Environ Microbiol 71: 4539–4547. 16085847 10.1128/AEM.71.8.4539-4547.2005 PMC1183363

[39] Hahn MW, Lang E, Brandt U, Wu QL, Scheuerl T (2009) Emended description of the genus *Polynucleobacter* and the species *P. necessarius* and proposal of two subspecies P *necessarius* subspecies *necessarius* subsp. nov. and *P. necessarius* subsp. *asymbioticus* subsp. nov. Int J Syst Evol Microbiol 59: 2002–2009. 19567561 10.1099/ijs.0.005801-0 PMC2956138

[40] Wu QL, Hahn MW (2006) High predictability of the seasonal dynamics of a species-like *Polynucleobacter* population in a freshwater lake. Environ Microbiol 8: 1660–1666. 16913925 10.1111/j.1462-2920.2006.01049.x

[41] Hahn MW, Scheuerl T, Jezberová J, Koll U, Jezbera J, et al. (2012) The passive yet successful way of planktonic life: genomic and experimental analysis of the ecology of a free-living *Polynucleobacter* population. PLoS One 7(3), e32772. 10.1371/journal.pone.0032772 PMC3308952 22448227

[42] Boyer SL, Flechtner VR, Johansen JR (2001) Is the 16S–23S rRNA internal transcribed spacer region a good tool for use in molecular systematics and population genetics? A case study in cyanobacteria. Mol Biol Evol 18: 1057–1069. 11371594 10.1093/oxfordjournals.molbev.a003877

[43] Risso C, Sun J, Zhuang K, Mahadevan R, DeBoy R, et al (2009) Genome-scale comparison and constraint-based metabolic reconstruction of the facultative anaerobic Fe(III)-reducer *Rhodoferax ferrireducens* . BMC Genomics 10: 447. 19772637 10.1186/1471-2164-10-447 PMC2755013

[44] Stewart FJ, Cavanaugh CM (2007) Intragenomic variation and evolution of the internal transcribed spacer of the rRNA operon in bacteria. J Mol Evol 65: 44–67. 17568983 10.1007/s00239-006-0235-3

[45] Wu QL, Peng X, Wen-Tso L (2010) East Tibetan Lakes Harbour Novel Clusters of *Picocyanobacteria* as Inferred from the 16S–23S rRNA Internal Transcribed Spacer Sequences. Microb Ecol 59: 614–622. 19904569 10.1007/s00248-009-9603-z

[46] Hoffmann M, Brown EW, Feng PC, Keys CE, Fischer M, et al (2010) PCR-based method for targeting 16S–23S rRNA intergenic spacer regions among *Vibrio* species. BMC Microbiol 10: 90 doi:10.1186/1471-2180-10-90 20331883 10.1186/1471-2180-10-90 PMC2856557

[47] Jezbera J, Jezberová J, Brandt U, Hahn MW (2011) Ubiquity of *Polynucleobacter necessarius* subspecies *asymbioticus* results from ecological diversification. Environ Microbiol 13: 922–931 doi:10.1111/j.1462-2920.2010.02396.x 21208356 10.1111/j.1462-2920.2010.02396.x PMC3087241

[48] Stackebrandt E, Ebers J (2006) Taxonomic parameters revisited: tarnished gold standards. Microbiology Today, 152–155.

[49] Galperin MY (2005) A census of membrane-bound and intracellular signal transduction proteins in bacteria: Bacterial IQ, extroverts and introverts. BMC Microbiol 5: 35. 15955239 10.1186/1471-2180-5-35 PMC1183210

[50] Yooseph S, Nealson KH, Rusch DB, McCrow JP, Dupont CL, et al (2010) Genomic and functional adaptation in surface ocean planktonic prokaryotes. Nature 468: 60–66. 21048761 10.1038/nature09530

[51] Giroldo D, Vieira AAH (2002) An extracellular sulphated fucose-rich polysaccharides produced by a tropical strain of *C. obovata* (*Cryptophyceae*). J Appl Phycol 14: 185–191.

[52] Giroldo D, Vieira AAH (2005) Polymeric and free sugars released by three phytoplanktonic species from a freshwater tropical eutrophic reservoir. Journal of Plankton Research 27: 695–705.

[53] Grossart HP, Levold F, Allgaier M, Simon M, Brinkhoff T (2005) Marine diatom species harbour distinct bacterial communities. Environ Microbiol 7: 860–873. 15892705 10.1111/j.1462-2920.2005.00759.x

[54] Horňák K, Jezbera J, Šimek K (2008) Effects of a *Microcystis aeruginosa* bloom and bacterivory on bacterial abundance and activity in a eutrophic reservoir. Aquat Microb Ecol 52: 107–117.

[55] Alonso C, Zeder M, Piccini C, Conde D, Pernthaler J (2009) Ecophysiological differences of betaproteobacterial populations in two hydrochemically distinct compartments of a subtropical lagoon. Environ Microbiol 11: 867–876. 19040452 10.1111/j.1462-2920.2008.01807.x

[56] Boenigk J, Stadler P, Wiedlroither A, Hahn MW (2004) Strain-specific differences in the grazing sensitivities of closely related ultramicrobacteria affiliated with the *Polynucleobacter* cluster. Appl Environ Microbiol 70: 5787–5793. 15466515 10.1128/AEM.70.10.5787-5793.2004 PMC522116

[57] Vannini C, Pöckl M, Petroni G, Wu QL, Lang E, et al (2007) Endosymbiosis in statu nascendi: Close phylogenetic relationship between obligately endosymbiotic and obligately free-living *Polynucleobacter* strains (*Betaproteobacteria*). Environ Microbiol 9: 347–359. 17222133 10.1111/j.1462-2920.2006.01144.x

[58] Urbanczyk H, Ast JC, Higgins MJ, Carson J, Dunlap PV (2007) Reclassification of *Vibrio fischeri*, *Vibrio logei*, *Vibrio salmonicida* and *Vibrio wodanis* as *Aliivibrio fischer*i gen. nov, comb. nov, *Aliivibrio logei* comb. nov, *Aliivibrio salmonicida* comb. nov. and *Aliivibrio wodanis* comb. nov. Int J Syst Evol Microbiol 57: 2823–2829. 18048732 10.1099/ijs.0.65081-0

[59] Manz W, Amann R, Ludwig W, Wagner M, Schleifer K-H (1992) Phylogenetic oligodeoxynucleotide probes for the major subclasses of Proteobacteria: problems and solutions. Syst Appl Microbiol 15: 593–600.

[60] Weisburg WG, Barns SM, Pelletier DA, Lane DJ (1991) 16S ribosomal DNA amplification for phylogenetic study. J Bacteriol 173: 697–703. 1987160 10.1128/jb.173.2.697-703.1991 PMC207061

[61] Lane DJ, Pace B, Olsen GJ (1985) Rapid determination of 16S ribosomal RNA sequences for phylogenetic analyses. Proc Natl Acad Sci USA 82: 6955–6959. 2413450 10.1073/pnas.82.20.6955 PMC391288

[62] Fisher MM, Triplett EW (1999) Automated approach for ribosomal intergenic spacer analysis of microbial diversity and its application to freshwater bacterial communities. Appl Environ Microbiol 65: 4630–4636. 10508099 10.1128/aem.65.10.4630-4636.1999 PMC91617

[63] Katoh K, Misawa K, Kuma K, Miyata T (2002) MAFFT: a novel method for rapid multiple sequence alignment based on fast Fourier transform. Nucleic Acids Res 30: 3059–3066. 12136088 10.1093/nar/gkf436 PMC135756

[64] Hall TA (1999) BioEdit: a user-friendly biological sequence alignment editor and analysis program for Windows 95/98/NT. Nucl Acids Symp Ser 41: 95–98.

[65] Tamura K, Peterson D, Peterson N, Stecher G, Nei M, et al (2011) MEGA5: Molecular Evolutionary Genetics Analysis using Maximum Likelihood, Evolutionary Distance, and Maximum Parsimony Methods. Mol Biol Evol 28: 2731–2739. 21546353 10.1093/molbev/msr121 PMC3203626

[66] Posada D (2008) jModelTest: Phylogenetic Model Averaging. Mol Biol Evol 25: 1253–1256. 18397919 10.1093/molbev/msn083

[67] Guindon S, Gascuel O (2003) A simple, fast, and accurate algorithm to estimate large phylogenies by maximum likelihood. Syst Biol 52: 696–704. 14530136 10.1080/10635150390235520

[68] Huelsenbeck JP, Ronquist F (2001) MRBAYES: Bayesian inference of phylogeny. Bioinformatics 17: 754–755. 11524383 10.1093/bioinformatics/17.8.754

[69] Ludwig W, Strunk O, Westram R, Richter L, Meier H, et al (2004) ARB: a software environment for sequence data. Nucleic Acids Res 32(4): 1363–1371. 14985472 10.1093/nar/gkh293 PMC390282

[70] Posch T, Pernthaler J, Alfreider A, Psenner A (1997) Cell-specific respiratory activity of aquatic bacteria studied with the tetrazolium reduction method, Cyto-clear slides, and image analysis. Appl Environ Microbiol 63: 867–873. 16535553 10.1128/aem.63.3.867-873.1997 PMC1389118

[71] Posch T, Franzoi J, Prader M, Salcher MM (2009) New image analysis tool to study biomass and morphotypes of three major bacterioplankton groups in an alpine lake. Aquat Microb Ecol 54: 113–126.

[72] Loferer-Krössbacher M, Klima J, Psenner R (1998) Determination of bacterial cell dry mass by transmission electron microscopy and densitometric image analysis. Appl Environ Microbiol 64: 688–694. 9464409 10.1128/aem.64.2.688-694.1998 PMC106103

