# Correction: The Diversity of the *Limnohabitans* Genus, an Important Group of Freshwater Bacterioplankton, by Characterization of 35 Isolated Strains

**DOI:** 10.1371/annotation/4fe772bd-a77f-4142-92c2-256be161f263

**Published:** 2013-09-12

**Authors:** Vojtěch Kasalický, Jan Jezbera, Martin W. Hahn, Karel Šimek

A grant and two funding organizations were incorrectly omitted from the Funding Statement. The Funding Statement should read: "This study was largely supported by the Grant Agency of the Czech Republic under research grants 206/08/0015 (granted to KS) and P504/10/0566 (granted to JJ), the institutional project of the ASCR No. AV0Z 60170517, and the project Postdok_BIOGLOBE (CZ.1.07/2.3.00/30.0032) co-financed by the European Social Fund and the state budget of the Czech Republic. The authors also profited from the Czech-Austrian mobility found KONTAKT projects MEB 060602/CZ 05-2007 (granted to KS and MWH) and MEB 060901/WTZ project 406600 (granted to JJ and MWH). The funders had no role in study design, data collection and analysis, decision to publish, or preparation of the manuscript." 

